# Feasibility, acceptability, and cost of tuberculosis testing by whole-blood interferon-gamma assay

**DOI:** 10.1186/1471-2334-6-47

**Published:** 2006-03-15

**Authors:** Puneet Kumar Dewan, Jennifer Grinsdale, Sally Liska, Ernest Wong, Robert Fallstad, L Masae Kawamura

**Affiliations:** 1 Division of Tuberculosis Elimination, Centers for Disease Control and Prevention, Atlanta, Georgia; 2 San Francisco Department of Public Health, San Francisco, California

## Abstract

**Background:**

The whole-blood interferon-gamma release assay (IGRA) is recommended in some settings as an alternative to the tuberculin skin test (TST). Outcomes from field implementation of the IGRA for routine tuberculosis (TB) testing have not been reported. We evaluated feasibility, acceptability, and costs after 1.5 years of IGRA use in San Francisco under routine program conditions.

**Methods:**

Patients seen at six community clinics serving homeless, immigrant, or injection-drug user (IDU) populations were routinely offered IGRA (Quantiferon-TB). Per guidelines, we excluded patients who were <17 years old, HIV-infected, immunocompromised, or pregnant. We reviewed medical records for IGRA results and completion of medical evaluation for TB, and at two clinics reviewed TB screening logs for instances of IGRA refusal or phlebotomy failure.

**Results:**

Between November 1, 2003 and February 28, 2005, 4143 persons were evaluated by IGRA. 225(5%) specimens were not tested, and 89 (2%) were IGRA-indeterminate. Positive or negative IGRA results were available for 3829 (92%). Of 819 patients with positive IGRA results, 524 (64%) completed diagnostic evaluation within 30 days of their IGRA test date. Among 503 patients eligible for IGRA testing at two clinics, phlebotomy was refused by 33 (7%) and failed in 40 (8%). Including phlebotomy, laboratory, and personnel costs, IGRA use cost $33.67 per patient tested.

**Conclusion:**

IGRA implementation in a routine TB control program setting was feasible and acceptable among homeless, IDU, and immigrant patients in San Francisco, with results more frequently available than the historically described performance of TST. Laboratory-based diagnosis and surveillance for *M. tuberculosis *infection is now possible.

## Background

Providers in the United States and Europe have for many years relied on the tuberculin skin test (TST) to detect infection with *Mycobacterium tuberculosis *in patients. The limitations of the TST are well documented, including placement variability, inter-reader variability, boosting, and difficulty in interpreting results in patients previously vaccinated with bacille Calmette Guerin (BCG) or with non-tuberculosis mycobacterial (NTM) infection. [1,2] Patients may find the test inconvenient because they must return in 48–72 hours for result reading and interpretation. TST use poses operational challenges, such as the training and retraining of numerous health care workers in proper test performance and interpretation. Patients and providers alike may have low confidence in test results, particularly when interpreting results for patients with prior BCG vaccination. Furthermore, the decentralized nature of the TST makes collection of valid population-based surveillance data for *M. tuberculosis *infection very challenging, particularly as latent TB infection (LTBI) is not a reportable medical condition in most areas.

Laboratory-based testing for *M. tuberculosis *infection could in theory overcome the technical and operational limitations of the TST, but until recently no such test was commercially available in the United States. However, in November 2001, the United States Food and Drug Administration approved the first blood assay for *Mycobacterium tuberculosis *for use as an aid in the diagnosis of *M. tuberculosis *infection. Commercially known as the QuantiFERON^®^-TB test (Cellestis International, Carnegie, Australia), this assay provides information about patients' cell-mediated immune response to *M. tuberculosis *by measuring interferon-γ produced in whole blood after incubation with purified protein derivative (PPD) from *M. tuberculosis *[3] Use of this interferon-γ release assay (IGRA) for detection of *M. tuberculosis *infection has been reported in several research studies and in small hospital- or laboratory-based implementations [4-8].

No field experiences using blood assays for *M. tuberculosis *testing in TB programs have been reported, and many unanswered questions remain about use under routine programmatic conditions. In November 2003, the San Francisco Department of Public Health (SFDPH) began limited implementation of IGRA use, substituting an IGRA for the TST in a TB testing and prevention program. SFDPH community health centers have routinely screened patients for TB using risk factor-based testing in accordance with CDC guidelines for targeted testing and treatment of LTBI. [9] Six clinical sites serving distinct patient populations with TB risk factors were selected to switch from the TST to IGRA as the routine test for infection with *M. tuberculosis*. These sites included two clinics serving a primarily homeless population (homeless clinics), two immigrant and refugee screening clinics (immigrant clinics), the county hospital methadone clinic, and the county TB clinic. TB testing procedures at these clinical sites were revised to specify that the IGRA was the default TB test offered to patients in whom TB testing was indicated.

We sought to evaluate 1) the feasibility of routine IGRA use for TB testing, as measured by valid IGRA results and completion of subsequent evaluation of patients with positive IGRA results, 2) the acceptability of blood assays for *M. tuberculosis *to patients, as measured by phlebotomy refusal, and 3) the costs of implementing IGRA testing for the health care system in San Francisco.

## Methods

### Program description

The San Francisco Department of Public Health (SFDPH) operates a network of 12 community health centers throughout San Francisco and a dedicated tuberculosis (TB) clinic at the county hospital. SFDPH community health centers have routinely screened patients for TB using risk-factor based screening in accordance with Centers for Disease Control and Prevention (CDC) guidelines for targeted testing and treatment of TB. [10] Briefly, patients identified with medical or epidemiologic risk factors for TB infection (including HIV infection, diabetes mellitus, current or planned immunosuppressive medications, homelessness, injection drug use, or immigration within 5 years from a TB-endemic country) were screened for TB at health centers by symptom review and a tuberculin skin test (TST). If either symptom screen or TST results were positive, then patients were referred to the TB clinic for chest radiograph (CXR), medical evaluation to exclude active TB, and consideration for treatment of latent tuberculosis infection (LTBI). Patients with prior treatment for TB infection or prior positive TST results were not evaluated by TST, but instead were directly referred to TB clinic for CXR and medical evaluation. If patients with positive TST results did not arrive at the SFDPH TB clinic within 30 days of referral, SFDPH routinely notified the referring clinic of the incomplete evaluation, and requested that the patient be again referred for medical evaluation. This retrospective program evaluation was undertaken with the ethical approval of the Centers for Disease Control and Prevention.

### Implementation of whole-blood interferon-gamma assay screening

Six clinical sites serving distinct patient populations with TB risk factors were chosen to switch from the TST to a blood assay for *Mycobacterium tuberculosis *(BAMT) as the routine test for TB infection. The BAMT chosen for use was the Quantiferon^®^-TB (Cellestis International, Carnegie, Australia), an interferon gamma release assay (IGRA) which at the time was the only BAMT approved for use by the Food and Drug Administration. These sites included 2 clinics serving a primarily homeless population (homeless clinics), 2 immigrant and refugee screening clinics (immigrant clinics), the county hospital methadone clinic, and the county TB clinic. TB screening procedures at these clinical sites were revised so that the IGRA was the default TB screening test offered to patients. In accordance with CDC guidelines for the Quantiferon^®^-TB assay, [3] providers were instructed to use the IGRA for all patients who were >17 years of age, not a contact to a TB case, not pregnant, and not immunocompromised; if the patient had any of these IGRA exclusion criteria, a TST was used. Because of same-day specimen processing requirements for the IGRA, providers were also instructed to use the TST instead if patients refused phlebotomy, if phlebotomy yielded less than 4 ml blood, or if the daily courier had already taken specimens to the laboratory. Patients were asked to return to clinic after 3 working days for IGRA results; those with positive IGRA results were managed in the same manner as patients with positive TST results, i.e. referred to TB clinic for radiograph and medical evaluation.

### Laboratory methods

Specimens were processed and evaluated by IGRA according to manufacturer instructions[11] At the time of this evaluation, this was the only IGRA for tuberculosis screening commercially available in the United States. Specimens were sent via courier to the laboratory for processing on the same day as phlebotomy. Briefly, 1 ml aliquots of heparinized whole-blood were incubated with 3 drops saline (nil), phytohemaglutinin (mitogen control), 5 ug/ml PPD from M. tuberculosis (tuberculin), and 5 ug/ml PPD from M. avium (avian sensitin). Specimens were incubated for 12 hours at 37°C. Plasma was collected and IFN-G concentration was determined by ELISA provided with the kit.

IGRA results were interpreted based on the proportion of IFN-G released in response to tuberculin, compared with mitogen, accounting for the background IFN-G level in the nil control samples [(tuberculin-nil)/(mitogen-nil) × 100 = percentage tuberculin response]. The percentage avian difference was calculated as [(avian-nil) - (tuberculin-nil)/(mitogen-nil) × 100]. An IGRA result was indeterminate if the (mitogen-nil) IFN-G level ≤ 1.5 IU. An IGRA result was negative if the (mitogen-nil) was ≥ 1.5 IU IFN-G and the percentage tuberculin response ≤ 15%. An IGRA result was positive if the following criteria were met: 1) (mitogen-nil) and (tuberculin-nil) ≥ 1.5 IU IFN-G, 2) percentage tuberculin response ≥ 15%, and 3) percentage avian difference ≤ 10%. Although manufacturer guidelines stipulated a 'conditional positive' interpretation for low-risk individuals, conditional positive results were not separately reported because SFDPH did not screen low-risk individuals. [3]

### Cohort review of patients with IGRA results

IGRA results from patients screened from November 2003 – February 2005 were reviewed. All patients for whom a specimen was received in the laboratory were included, even if that specimen could not be tested for technical reasons. Patient results were stratified by age and screening site. As the evaluation used data collected under routine programmatic conditions, health information beyond age and epidemiologic TB risk factor was not collected. We included only the first test result for each patient in the cohort, as some patients were screened more than once during the 15-month evaluation period. In the small subset of patients with IGRA results who were suspected of having active TB after their TB clinic medical evaluation (American Thoracic Society classification TB-5), [2] we evaluated the accuracy of the IGRA for the diagnosis of active TB. We reviewed TB clinic medical records to determine the outcome of patients classified as TB suspects who had IGRA results reported within 2 weeks of initiation of anti-TB treatment. Standard case definitions for a report of a verified case of tuberculosis (RVCT) were used. [12] Medical records were also reviewed for available TST results from within 30 days of the IGRA date

### Acceptability of blood-test based TB screening

To evaluate acceptability, we reviewed TB screening logs that had been implemented at 2 of the 6 clinic sites. The TB screening logs recorded the experience of all patients consecutively screened for TB by IGRA or TST at a homeless clinic from May through November 2004 (n = 406), and at a refugee clinic from June through December 2004 (n = 145). This patient population only partially overlapped with the cohort of patients with IGRA test results, as it also included patients evaluated by TST, hence has been presented as a parallel but separate evaluation.

All patients seen for TB screening at these clinics were routinely offered IGRA unless they belonged to populations for whom the IGRA was not recommended. Patients who were not offered IGRA, who refused phlebotomy for IGRA, or who had unsuccessful phlebotomy were offered TST instead. Logs were reviewed to identify instances of TB screenings where patients refused phlebotomy for IGRA, had unsuccessful phlebotomy for IGRA, or fell into an IGRA exclusion category. Patients with positive TST, IGRA, or symptom review results from these clinic were routinely referred to the SFDPH TB Clinic for further medical evaluation. We reviewed TB clinic medical records to determine the proportion of these patients with positive IGRA results who had a CXR and medical evaluation within 30 days of their IGRA test date.

### Cost assessment

We conducted a cost assessment from the perspective of the health care system in San Francisco to quantify the expenditures necessary to implement interferon-gamma assay TB screening. All clinical and laboratory supplies needed to perform the test were measured. Because the test is performed most efficiently in batches of 20  specimens and has been performed in this way in San Francisco, we  recorded the minimum time necessary for laboratory staff to complete  testing on one batch of specimens. Laboratory supplies used for a single batch of 20 patient specimens was recorded. Staff costs for clinical and laboratory staff were calculated using SFDPH hourly salary tables for mid-grade employees, with 26% added to account for employee benefits. Training sessions for clinical staff were not included in costs, as this was judged to be a one-time start-up activity of the TB control program. We used retail costs for all supplies including the commercial kits for the interferon gamma assay; though SFDPH sometimes gets discounts for supplies through vendors, these discounts are variable. Shipping charge costs were drawn from a SFDPH contract with a local courier service. Laboratory equipment costs were assigned a per-patient cost by determining the proportion of daily time used for a batch of 20 specimens, with 3% linear discounting applied over an estimated 10-year equipment lifespan.

## Results

### Results of IGRA testing

From November 2003 through February 2005, SFDPH evaluated 4143 unique patients by IGRA at six clinic sites. The median patient age was 43.4 years (range 2–100); this included 79 (1.9%) patients <18 years old, despite the recommendation that patients under 18 not be tested. Among the 4143 patients evaluated by IGRA, 225 (5.4%) had specimens rejected by the laboratory because of insufficient blood, clotted blood, or receipt after cut-off time for daily specimen processing. Among the remaining 3918 specimens tested, 2600 (66%) were submitted by the two homeless clinics.

Of the 3918 specimens tested results were positive in 819 (21%), negative in 3010 (77%), and indeterminate in 89 (2%). (Table 1) Patients tested at the methadone clinic were more likely to have indeterminate results than those at all other sites combined (relative risk [RR] 3.5, 95% confidence interval (CI) 2.0–6.1). IGRA results from 819 (21%) were positive. A higher proportion of patients had positive IGRA results at the immigrant and refugee clinics (34%) and the TB clinic (33%) than at the homeless clinics (16%) or methadone clinic (17%). Among the 819 patients with positive IGRA results, 524 (64%) subsequently completed diagnostic evaluation at the SFDPH TB Clinic within 30 days of IGRA testing, 575 (70%) within 60 days, and 593 (72%) within 90 days (Table 2). A higher proportion of patients tested at the TB clinic completed diagnostic evaluation than patients seen at any other clinic site. We were unable to compare the evaluation outcomes of patients tested by TST, as TST results have never been routinely reported in San Francisco.

**Table 1:** Results from routine testing of patients with risk factors for *M. tuberculosis *infection using a PPD-based whole blood interferon-γ release assay (IGRA), stratified by testing site. San Francisco, November 2003 – February 2005

	Homeless Clinics (n = 2)	TB Clinic	Immigrant & Refugee (n = 2)	Methadone Clinic	Total
IGRA Result	n (%)	n (%)	n (%)	n (%)	N (%)

Positive	411 (16)	213 (33)	164 (34)	31 (17)	819 (21)
Negative	2148 (82)	419 (64)	303 (63)	140 (76)	3010 (77)
Indeterminate	41 (2)	21 (3)	14 (3)	13 (7)	89 (2)

Total tested	2600	642	481	184	3918

**Table 2:** Cumulative proportion of patients with positive IGRA results with documented completion of chest radiograph and medical evaluation, by testing site. Days counted from blood draw date.

	Homeless Clinics (n = 2)	TB Clinic	Immigrant & Refugee (n = 2)	Methadone Clinic	Total
Evaluation completed by	n = 411 (%)	n = 212 (%)	n = 164 (%)	n = 31 (%)	N = 818 (%)

30 days	245 (60)	165 (78)	94 (57)	19 (61)	524 (64)
60 days	267 (65)	181 (85)	105 (64)	21 (68)	575 (70)
90 days	274 (67)	191 (90)	107 (65)	21 (68)	593 (72)

During the evaluation period, SFDPH identified a total of 352 patients as suspected of having TB (ATS classification TB-5), but only 20 (5.7%) had IGRA results available from 30 days prior to and until 14 days after the initiation of anti-TB treatment. Nine (45%) of the 20 TB suspects with IGRA results were diagnosed with a verified case of TB, including seven laboratory-confirmed and two clinically verified TB cases. Although this sample was not large enough to allow assessment of IGRA accuracy for TB diagnosis, among the seven laboratory-confirmed TB cases, two had negative IGRA results. No TST results were available for comparison.

### Acceptability of blood test-based TB testing

From May through November 2004, one homeless clinic examined 406 unique patients (Table 3); 31 (8%) belonged to populations for whom IGRA was not recommended, and were offered TST instead. Among the 375 eligible for IGRA testing, 312 (83%) had a specimen sent for IGRA testing; phlebotomy failed in 33 (9%) and was refused by 30 (8%). In the 312 patients with IGRA performed, 310 (99%) had interpretable (i.e., positive or negative) results. A historical comparison of the TST reading return rate was available from the routine records of the same clinic. Before IGRA implementation in 2003, 1523 (88%) of 1734 patients tested by TST had returned for TST reading within 4 days. Of note, this clinic serves clients who frequently needed "TB clearance" for homeless shelters, and has a historically high TST return rate.

**Table 3:** Acceptability of blood-based TB test at a homeless clinic and a  refugee-screening clinic, San Francisco, May-December 2004. *Patients  less than 18 years old, HIV-infected, immunocompromised, or pregnant  were ineligible for IGRA. **Seen at SFDPH TB clinic for medical  evaluation and chest radiograph within 30 days of IGRA date.

	Homeless Clinic n (%)	Refugee Clinic n (%)	Total N (%)
Tested by TST or IGRA	406	145	551
IGRA Ineligible*	31 (8)	17 (12)	48 (9)
IGRA eligible	375 (100)	128 (100)	503 (100)
IGRA performed	312 (83)	113 (88)	425 (84)
After lab time cut-off	0 (0)	5 (4)	5 (1)
Phlebotomy refused	30 (8)	3 (2)	33 (7)
Phlebotomy failed	33 (9)	7 (5)	40 (8)
TST attempted	94 (100)	32 (100)	126 (100)
Returned for reading	68 (72)	32 (100)	100 (79)
Positive IGRA results	53 (100)	50 (100)	103 (100)
Successfully evaluated**	42 (79)	34 (68)	76 (74)

From June through December 2004, the refugee clinic examined 145 unique patients; 17 (12%) belonged to populations for whom IGRA was not recommended, and these patients were offered TST instead. Among the 128 patients eligible for IGRA, phlebotomy failed in seven (5%) and was refused by three (2%). In the 113 patients with IGRA performed, 108 (96%) had interpretable results. From this clinic, no historical information about patient return for TST reading was available for comparison.

### Cost assessment

We estimated the cost to the health care system in San Francisco per patient tested by IGRA as $33.67 (Table 4). Most of this cost ($27.91, 83%) was incurred by the laboratory, with a smaller proportion incurred during phlebotomy and shipping of specimens. Almost half of the total cost was the commercial kit itself, but $8.91 (26%) of the per-patient cost was attributable to laboratory staff costs. Owing to the pipetting of variable volumes and result interpretation required for the commercial test, federal laboratory regulations indicate that a clinical laboratory scientist perform the test instead of a lower salary rate laboratory technician.

**Table 4:** Health care system costs for use of whole-blood interferon-γ assay (QuantiFERON-TB^®^) for testing for *M. tuberculosis *infection. Total costs incurred for batch of 20 patients tested (2004$). Equipment usage based on time used during assay, with 10-year estimated lifespan for all laboratory equipment. Laboratory staff costs based on hourly salary plus benefits cost for mid-grade staff.

Task Group	Resources Used	Units Used per Batch of 20 Patients	Cost Per Patient Tested ($)
Phlebotomy	Nursing staff time	1.34 hours (4 minutes per patient)	3.06
	Supplies	20 tubes and needles	2.20
Shipping	Courier service	1 daily courier trip ($10)	0.50
Laboratory	Commercial kits	0.5 commercial kits	16.50
	Consumables	Laboratory supplies	2.39
	Equipment usage	Orbital rocker, ELISA washer and reader, incubator, hood, computer	0.11
	Clerical staff	0.66 hours	0.77
	Clinical lab scientist	4.16 hours	8.14

Total			$33.67

## Discussion

These are the first reported operational outcomes of program efforts to switch from the TST to a blood assay for *M. tuberculosis*. In San Francisco, TB testing using the IGRA was feasible and acceptable under routine programmatic conditions in several different clinics. We tested 4143 patients by IGRA and obtained usable results on 3829 (92%). Indeterminate results were uncommon (2%). The PPD-based IGRA used during this time period has since been superseded by newer tests using *Mycobacterium tuberculosis*-specific region of difference 1 (RD1) antigens (such as early secretory antigenic target 6 and culture filtrate protein 10) for T cell stimulation. Available evidence suggests that these RD1-based IGRA tests are more specific than the TST, and have similar or slightly higher sensitivity in most situations[13] But the operational experiences we describe remain relevant to any public or occupational health program currently using the TST and considering use of a blood assay for *M. tuberculosis*.

IGRA results were available to the TB program on 92% of persons tested, representing a likely improvement in TB testing outcomes compared to TST. This outcome compares favorably to the reported return rate for TST reading in similar patient populations in other cities, including 84% at a needle-exchange program in Baltimore, 64% at a public health program in Atlanta, and 47% in a street outreach program for drug users in Long Beach. [14-16] Since results are available on most patients tested by IGRA, program resources may be directed to improving follow-up of patients with positive IGRA results. Ample room remains for follow-up improvement; among the 819 patients with positive results, subsequent medical and radiograph evaluations were completed within 30 days in 524 (64%). Collection of comparable outcomes for TST was not possible from program data in San Francisco, since no surveillance system existed for TST results.

Surveillance for new instances of infection with *M. tuberculosis *has been previously attempted in San Francisco, but failed due to the decentralized and subjective nature of the TST and to the large resource investment required to maintain surveillance system function in hundreds of testing locations throughout the city. Laboratory-based testing allowed us to obtain accurate data for surveillance of LTBI from these IGRA pilot sites, including both denominator data for patients tested and longitudinal data alerting SFDPH to instances of newly detected *M. tuberculosis *infection, including the occurrence of IGRA 'conversion', i.e. positive results in a patient with previously negative results. Furthermore, the use of a laboratory-based test improved information availability for community providers and likely reduced duplicate testing, as previous results were available via an electronic laboratory result reporting system accessible to many community clinics. In San Francisco, since March 2005 a newer version of the IGRA (QuantiFERON-TB Gold^®^, Cellestis International), which uses RD-1 antigens for leukocyte stimulation, has been available to all SFDPH clinics and key community organizations providing services to homeless and immigrant populations. Through wider use of this newer IGRA, we have essentially created a laboratory-based surveillance system for infection with *M. tuberculosis*, with reliable reporting of newly detected instances of infection with *M. tuberculosis *among key populations such as the homeless, IDUs, and new immigrants. This advance in TB surveillance capacity may allow SFDPH to more accurately target case finding and prevention activities in the future.

Phlebotomy for IGRA-based TB testing proved acceptable among a majority of patients at both a homeless clinic and a refugee clinic. Phlebotomy refusal was uncommon (8%), and phlebotomy failed in only 8% of patients with a high prevalence of intravenous drug use. At the homeless clinic, among patients tested by IGRA, a positive or negative result was obtained in 99%, which is greater than the proportion of patients who returned for TST reading at that particular site prior to IGRA implementation (88%).

IGRA implementation cost the health care system in San Francisco approximately $33.89 per patient tested, most of which (83%) was incurred by the laboratory. This cost assessment includes only measurable direct costs of phlebotomy, specimen transport, and laboratory specimen processing. Assuming all other costs remain the same, we have projected that the more-expensive RD1-based IGRA, QuantiFERON^®^-TB Gold, will cost approximately $37.39 per patient tested. In comparison, the Center for Medicare Services has estimated the cost of the TST to be $9.79. [17] Despite this lower per-test cost for TST, several factors such as differences in test specificity, patient time requirements, and the potential to lower the laboratory cost for IGRA through automation raise the possibility that the IGRA may be the more cost-effective test. Future cost-effectiveness analysis comparing IGRA and TST use in TB testing will be necessary, and should include potential cost savings from improved patient evaluation rates and avoidance of treatment of patients with false-positive TST results.

Several operational challenges were noted during IGRA implementation. Same-day processing for specimens necessitated early and often inconvenient cut-off times for specimen collection, as well as use of a courier service. Patients attending afternoon or evening clinics were forced to return another morning for IGRA testing or to have a TST. Future versions of the IGRA that might allow more flexible timing of specimen collection and transport would mitigate this challenge. The occasional occurrence of discordant IGRA and TST results proved time-consuming for patients and providers to manage. Programs implementing IGRA should establish guidelines to help providers manage such events.

Our operational evaluation has several limitations. We did not randomize patients or sites to TST or IGRA. A randomized study would yield a more accurate comparison of acceptability and testing outcomes. Our evaluation was not intended to evaluate IGRA concordance with TST results; studies addressing that question have been reported elsewhere[13] In accordance with CDC guidelines we excluded many patient populations such as children and HIV-infected persons from IGRA, but operational outcomes may differ in those groups. Patients with positive IGRA results may not have responded to SFDPH attempts to contact them, and instead may have been evaluated by private providers. Hence we may have underestimated the proportion of patients who were tested for TB after receiving positive IGRA results. Our cost estimates are specific for the health care system in San Francisco, and should not be considered generalizable. We did not collect data about costs incurred by patients, which may significantly differ between IGRA and TST. We used phlebotomy refusal to substitute for specific questions to patients about TST or IGRA preference. In our evaluation we did not extend data collection to include completion of treatment for LTBI, which would be the optimal outcome measure for feasibility, but may vary widely by setting and patient population. Lastly, our evaluation included only the first-generation PPD-based IGRA, but the use of new antigens should not necessarily affect programmatic outcomes such as feasibility and acceptability, which are likely to be common among blood assays for *M. tuberculosis*.

## Conclusion

IGRA-based TB testing was successfully implemented and well accepted under routine programmatic conditions. No adverse programmatic outcomes were identified. Laboratory-based surveillance for TB infection now appears possible. Programs considering switching from TST to IGRA may expect to realize benefits in completion rates of TB testing, reporting of results, and surveillance capacity. With improved RD1-based IGRA tests offering higher specificity than TST, programs may find that the avoidance of unnecessary testing and treatment of patients with false-positive TST results yields additional public health benefits.

## Competing interests

PKD is an employee for the US Centers for Disease Control and  Prevention, which has participated in clinical trials of the  Quantiferon^®^-TB test. The authors declare that they have no competing  interests.

## Authors' contributions

PKD designed, conducted, and analyzed the data from this evaluation, and also drafted the manuscript. JG provided data analysis support and critical comments for the manuscript. SL and EHW conducted the laboratory studies and provided critical comments for the manuscript. RF assisted with data entry and analysis. LMK contributed to the concept and design of the evaluation, and provided critical comments for the manuscript.

## Pre-publication history

The pre-publication history for this paper can be accessed here:


